# Successful Natalizumab Treatment of Two Female Individuals With Susac Syndrome

**DOI:** 10.1111/ene.70103

**Published:** 2025-03-03

**Authors:** Agni M. Konitsioti, Rafael Grajewski, Mark Schlamann, Michael Schroeter, Gereon R. Fink, Clemens Warnke

**Affiliations:** ^1^ Department of Neurology University of Cologne, Faculty of Medicine and University Hospital Cologne Cologne Germany; ^2^ Department of Ophthalmology Faculty of Medicine and University Hospital of Cologne Cologne Germany; ^3^ Department of Radiology and Neuroradiology, Faculty of Medicine and University Hospital University of Cologne Cologne Germany; ^4^ Cognitive Neuroscience, Institute of Neuroscience and Medicine (INM3) Research Center Jülich Jülich Germany; ^5^ Department of Neurology University Hospital Marburg Marburg Germany

**Keywords:** DMT, MRI, neuroimmunology, neuroinflammation, neuroopthalmology, susac, vasculitis

## Abstract

**Background:**

Susac syndrome is a rare autoimmune endotheliopathy that affects the central nervous system, retina, and inner ear, characterized by encephalopathy, branch retinal artery occlusions, and sensorineural hearing loss. Due to the heterogeneity of its presentation, early diagnosis, and treatment remain challenging.

**Objective/Methods:**

To evaluate the clinical outcomes and radiological responses in two patients with Susac syndrome treated with natalizumab in an off‐label therapeutic approach, clinical assessments and serial magnetic resonance imaging (MRI) were performed over a follow‐up period of up to 22 months to monitor disease progression and treatment response.

**Results:**

Both patients demonstrated clinical stabilization with reduced MRI and retinal angiography disease activity. Treatment was well tolerated, and no significant adverse events were reported during observation.

**Discussion:**

Natalizumab may constitute a potential off‐label therapeutic for Susac syndrome. Further studies are warranted to assess its efficacy and safety in this rare condition.

**Abbreviations**
ADEM: acute disseminated encephalomyelitis
AECA: anti‐endothelial cell IgG1 antibodies
BRAO: branch retinal artery occlusions
cMRI: Cranial MRI
CNS: central nervous system
FLAIR: Fluid‐attenuated inversion recovery
IVIG: intravenous immunoglobulins
JC virus: John Cunningham virus
MMF: mycophenolate mofetil
MRI: Magnetic resonance imaging
MS: multiple sclerosis
OCT: optical coherence tomography
SLE: systemic lupus erythematosus
SuS: Susac Syndrome
T1w FFE: T1‐weighted fast field echo
T2/TSE: T2‐weighted turbo spin echo
TPE: therapeutic plasma exchange
VLA‐4: very late antigen‐4

## 1 Introduction

Susac syndrome (SuS) is a rare neuroinflammatory disorder characterized by sensorineural hearing loss, encephalopathy, and branch retinal artery occlusions (BRAO). It predominantly affects women, with a female‐to‐male ratio of approximately 3.5:1, typically presenting in their third and fourth decades [1, 2]. Due to the disease rarity, epidemiological data are scarce, with an estimated annual incidence of 0.24 per 1,000,000 people in Central Europe [3].

Diagnosing SuS is particularly challenging, as the clinical triad is present in only 47% of the patients at disease onset, necessitating a high degree of clinical awareness for this rare disease [4]. SuS has several differential diagnoses, including multiple sclerosis (MS), systemic lupus erythematosus (SLE), acute disseminated encephalomyelitis (ADEM), migraine, Meniere's disease, viral and autoimmune encephalitis, and cerebral vasculitis [5, 6], owing to the overlapping symptoms and imaging features [7]. Diagnosis typically relies on MR imaging for callosal lesions [8], retinal angiography to assess BRAO, and vestibulocochlear function tests [8]. Formal diagnostic criteria have been established to enhance diagnostic accuracy [9]. The 2016 European Susac Consortium (EuSaC) criteria categorize SuS into three levels: definite SuS, where all three organs (brain, eye, and ear) are affected; probable SuS, with involvement of two organs; and possible SuS, where only one organ is affected [9].

The clinical course of SuS is highly variable, with episodic symptoms that may recur and lead to severe complications, for example, epilepsy, cognitive impairment, permanent hearing loss, and visual impairment [8]. Regarding the relapse rate in SuS patients, a recent review of 151 cases, classified as probable or definite SuS, reported relapses in 36 patients (24%) with a median time from diagnosis to relapse of 4 months [4]. In another retrospective analysis of medical records from an Australian cohort comprising 32 adult SuS patients retrospectively classified as definitive or probable SuS, clinical relapses occurred in 10 out of 22 patients (45%) initially treated for an alternative diagnosis and in three patients who had received no treatment for their initial alternative diagnosis [6].

Given the variable course and rarity of SuS, treatment is challenging, especially in refractory cases, compounded by the absence of objective biomarkers and the lack of randomized controlled trials that provide standardized treatment guidelines. Empirical treatment, including high‐dose corticosteroids, is recommended as initial treatment [10]. Evidence from expert guidelines, follow‐up of different cohorts of patients with SuS, and protocols for other autoimmune diseases with similar immunopathogenesis suggest potential benefits of immunosuppressive treatments such as azathioprine, cyclophosphamide, tacrolimus, mycophenolate mofetil (MMF), intravenous immunoglobulins (IVIG), rituximab, infliximab, and adjuvant therapies such as nimodipine or antiplatelet agents [8, 10]. In severe cases, therapeutic plasma exchange (TPE) has been used to alleviate relapse symptoms, although long‐term follow‐up data are lacking [11]. A CD8^+^ T‐cell‐mediated endotheliopathy has recently been suggested as a critical pathogenic mechanism in SuS, paving the way for more targeted therapeutic approaches such as the use of natalizumab [12]. Conversely, some reports indicate that SuS may not respond to immune therapies at all [13].

We present two cases of SuS with significant neurological symptoms that responded favorably to natalizumab. Based on these cases and other published reports, we propose that natalizumab should be systematically explored for SuS therapy, particularly in persons with a negative anti‐JC virus serology.

## 2 Case Presentation

### 2.1 Case Number 1. 25‐Old Female Individual With Spastic‐Ataxic Left Hemiparesis

**FIGURE 1:**
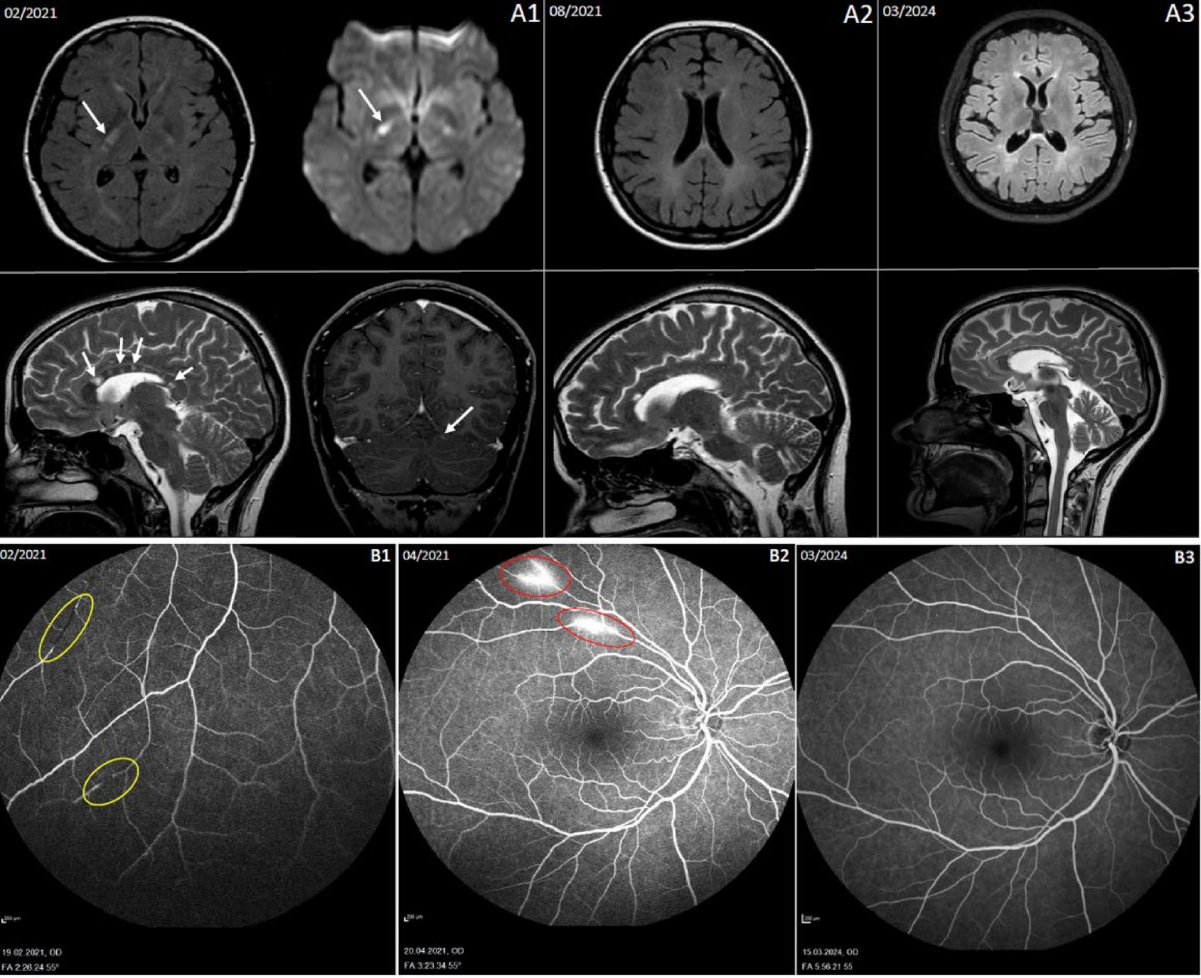
(A1) Cranial MRI scan from February 2021. Top panel: Axial fluid‐attenuated inversion recovery (FLAIR) sequence (left) and axial diffusion‐weighted imaging (DWI) (right). Bottom panel: Sagittal T2‐weighted turbo spin echo (T2/TSE) sequence (left) and coronal T1‐weighted fast field echo (T1w FFE SPIR) sequence with contrast agent (right). The imaging findings are indicative of an acute‐florid phase of a chronic inflammatory CNS disease, with a differential diagnosis of SuS syndrome, as evidenced by multiple corpus callosum lesions (white arrows, bottom panel, left) and diffusion‐restricted string‐of‐pearls‐like arranged lesions in the right posterior limb of the internal capsule (white arrows, top panel) and one cerebellar lesion (white arrows, bottom panel, right). (A2) Cranial MRI scan from August 2021. Top panel: Axial FLAIR sequence. Bottom panel: Sagittal T2/TSE sequence. Two months after initiation of natalizumab therapy, no new FLAIR lesions are observed, and the previously identified lesions remain broadly largely stable, with slight regression noted in some. No evidence of blood–brain barrier disruption or diffusion restriction is present. (A3) Cranial MRI scan from March 2024. Top panel: Axial FLAIR sequence. Bottom panel: Sagittal T2/TSE sequence. Eight months after the therapy pause (the last dose was administered in July 2023), there is no significant change compared to the previous examination from September 2023. Stable residual lesions are seen in the corpus callosum and periventricular regions, with no newly demarcated lesions detected. (B1) and (B2) Fluorescein angiography from February 2021 and April 2021 (prior to initiation of Natalizumab therapy): Vascular occlusions (yellow circles), ruptures, filling defects, and vasculitic enhancements in the optic nerve, with areas of leakage (red circles). (B3) Fluorescein angiography from March 2024 (following the last dose of natalizumab in July 2023): No evidence of inflammatory activity observed.

A 25‐year‐old female patient was urgently admitted to the hospital in February 2021 due to a subacute onset of spastic‐ataxic left hemiparesis 1 week before and bladder dysfunction persisting for 3 weeks. Additionally, she had migraine‐like headaches accompanied by visual disturbances with a central visual field defect in the left eye, which persisted for several weeks. In December 2020, she experienced an episode of vertigo and nausea, which improved with prednisolone. The cranial MRI scan showed numerous FLAIR‐hyperintense lesions with diffusion restriction in the corpus callosum and periventricular areas, along with string‐of‐pearls‐like lesions in the right posterior internal capsule (Figure 1A1). Cerebrospinal fluid (CSF) analysis revealed mild pleocytosis and elevated protein levels without intrathecal antibody synthesis. Ear, nose, and throat (ENT) evaluation revealed low‐frequency sensorineural hearing loss on the left side with normal brainstem auditory evoked potentials. The ophthalmological examination revealed retinopathy with pathological fluorescein angiography (Figure 1B1) and arcuate scotomas on perimetry. Extensive infectious and vasculitis diagnostics were unremarkable (Table S1). Based on these findings, we diagnosed SuS.

The patient was treated with a five‐day course of 1250 mg prednisolone per day (equals a glucocorticoid potency of 1 g/day methylprednisolone) with a tapering regimen (starting with 100 mg prednisolone followed by a reduction of 20 mg every other day) resulting in symptom improvement. Additionally, 100 mg of acetylsalicylic acid was prescribed. In April 2021, the patient experienced a relapse with new vascular occlusions and active retinal vasculitis (Figure 1B2). At that time, she was taking prednisolone at a daily dose of 25 mg. The subsequent ENT follow‐ups were unremarkable. The cranial MRI scan revealed a new FLAIR‐hyperintense lesion in the left pons without contrast enhancement. We initiated another three‐day course of high‐dose prednisolone with a tapering regimen as described above.

**FIGURE 2:**
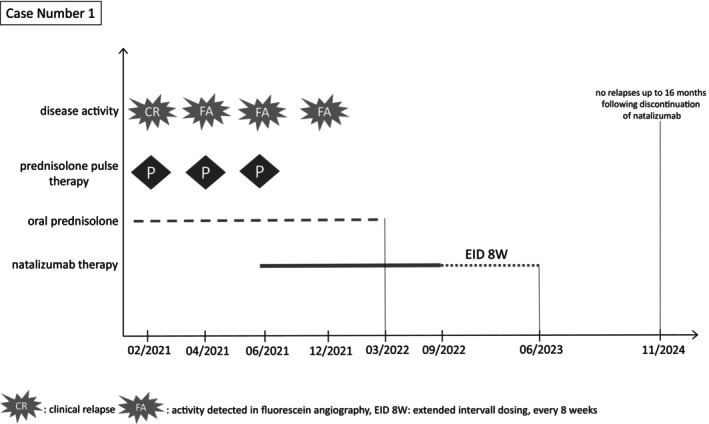
Illustration of the clinical progression, including treatment interventions and disease activity over time for case number 1.

The patient experienced another relapse in June 2021, with a clinically silent but angiographically active retinal vasculitis. A prednisolone pulse therapy was initiated, followed by a tapering regimen, and, after a negative JC‐virus antibody test, off‐label treatment with natalizumab (300 mg) was administered in combination with oral prednisolone therapy. Cranial MRI scans in August 2021 (Figure 1A2) and September 2022 demonstrated either stable or slightly regressed lesions. At the ophthalmological follow‐up in December 2021, fluorescein angiography revealed minimal peripheral residual activity in both eyes, prompting the continuation of oral prednisolone with 20 mg. Subsequent fluorescein angiographies in March 2022 and March 2023 showed no signs of inflammatory activity, allowing for the discontinuation of prednisolone. The follow‐up JC‐virus antibody tests remained negative. The cranial MRI scan in March 2023 revealed stable residual inflammatory lesions. Given the stable clinical course and imaging findings, natalizumab dosing was adjusted to an eight‐week interval in September 2022, with the final dose administered in July 2023, followed by a treatment‐free period. Follow‐up cranial MRI scans in September 2023 and March 2024 (Figure 1A3) and fluorescein angiography (Figure 1B3) showed stable findings, and no clinical relapses were reported up to 16 months following the discontinuation of natalizumab. A graphical representation illustrating the clinical progression, treatment interventions, and disease activity over time is provided in Figure 2.

### 2.2 Case Number 2. 28‐Year‐Old Female Patient With Hypoacusis and Visual Disturbances

In September 2022, a 28‐year‐old female was referred from the ENT department due to a two‐week history of bilateral hypoacusis with bilateral low‐frequency sensorineural hearing loss, visual disturbances, dizziness, and balance problems. Additionally, 4 weeks prior, she experienced a transient episode of left‐sided hemiparesis. A five‐day course of prednisolone pulse therapy (250 mg/day) in the ENT department improved audiometric results, though her symptoms persisted.

**FIGURE 3:**
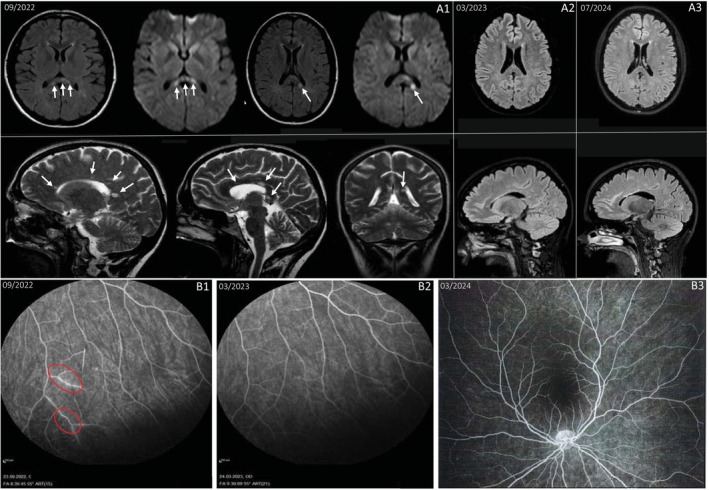
(A1) Cranial MRI scan from September 2022. Top panel: Axial Fluid‐attenuated inversion recovery (FLAIR) sequence and axial diffusion‐weighted imaging (DWI). Bottom panel: Axial and sagittal T2‐weighted turbo spin echo (T2/TSE) sequence: Multiple, predominantly diffusion‐restricted, round ‘snowball’ lesions are evident in the periventricular white matter, as well as the corpus callosum (arrows). (A2) Cranial MRI scan from March 2023 (3D FLAIR sequence). Six months following initiation of natalizumab therapy, there is an evident improvement of the previously observed lesions in the supratentorial white matter, with only smaller residual abnormalities. No evidence of blood–brain barrier disruption or new diffusion‐restricted lesions is present. No new lesions are identified. (A3) Cranial MRI scan from July 2024 (3D FLAIR sequence). Twenty two months post‐natalizumab therapy initiation, stable findings are observed with long‐term regression of supratentorial white matter lesions compared to September 2022. (B1) Fluorescein angiography from September 2022 (prior to initiation of natalizumab therapy): Vasculitic changes in the optic nerve, with areas of leakage indicated (red circles). (B2, B3) Fluorescein angiography from March 2023 and April 2024 (6 and 18 months after starting natalizumab therapy): No signs of inflammatory activity detected.

A cMRI revealed multiple new round lesions in the corpus callosum with characteristic ‘snowball’ appearances, along with periventricular FLAIR hyperintensities and diffusion abnormalities (Figure 3A1). CSF analysis showed normocytosis and slight protein elevation without intrathecal antibody synthesis. Infectious and vasculitis diagnostics were unremarkable (Table S1). Ophthalmological exams, including fluorescein angiography, identified multiple vascular occlusions, ruptures, bilateral filling defects, and vasculitic enhancements in both optic nerves, some with leakage (Figure 3B1). These findings led to a diagnosis of SuS.

Following a five‐day course of prednisolone pulse therapy with a tapering regimen, as described in case 1, the patient showed no symptom improvement. Given the severe clinical presentation and the patient's age, we initiated treatment with natalizumab and oral prednisolone in September 2022, following a negative JC‐virus antibody test. While dizziness had initially worsened during steroid therapy, her condition stabilized after the first natalizumab infusion. Besides abnormal bilateral tone audiometry, no neurological deficits persisted.

At ophthalmological follow‐up in November 2022, both eyes showed significant improvement, with reduced Susac‐typical vascular wall staining, leakage, and perfusion disturbances. However, some residual activity and leakage persisted in the left eye, so oral prednisolone therapy with a maintenance dose of 5 mg was continued. A short‐term follow‐up during oral prednisolone therapy showed a stable macular optical coherence tomography (OCT) and fluorescein angiography without signs of inflammatory activity, leading to the discontinuation of prednisolone. A cMRI in March 2023 (Figure 3A2) showed near‐complete resolution of the previously diffusion‐impaired white matter lesions, with only a few small residuals. Ophthalmological follow‐up in March 2023 revealed no vascular or inflammatory abnormalities on angiography (Figure 3B2). In April 2023, the patient experienced a relapse with balance disturbances, which improved after 3 days of prednisolone pulse therapy. The cMRI findings remained unchanged.

**FIGURE 4:**
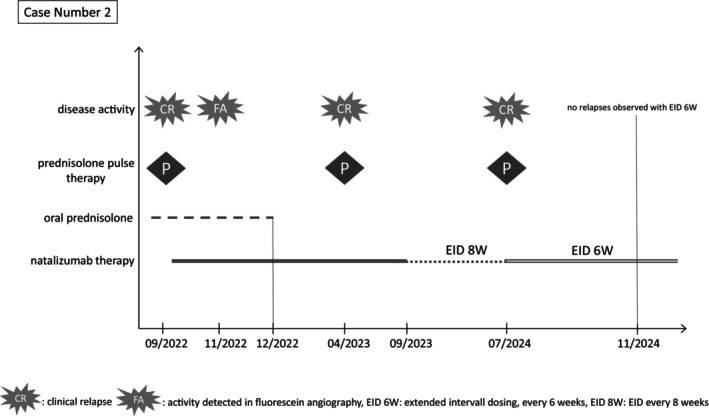
Illustration of the clinical progression, including treatment interventions and disease activity over time for case number 1.

One year after initiating natalizumab therapy (i.e., in September 2023) and due to stable disease, natalizumab dosing was adjusted to an eight‐week interval. The cMRI in October 2023 showed no new cerebral lesions. Ophthalmological follow‐up in March 2024 revealed no abnormalities on angiography (Figure 3B3). However, in July 2024, the patient had another relapse with new right‐sided tinnitus, balance disturbances, subjective left arm weakness, and transient bilateral visual disturbances. The cMRI (Figure 3A3) showed again stable supratentorial white matter changes 22 months post‐natalizumab therapy initiation. After 3 days of prednisolone pulse therapy, her symptoms began to improve. Given the increased clinical relapse activity observed following the extension of natalizumab dosing to eight‐week intervals, we pragmatically recommended returning to a six‐week dosing interval to optimize disease control, as trough serum natalizumab concentration and α4‐integrin saturation measurements were not available. Since then, no clinical disease activity has (yet) reoccurred. A follow‐up JC‐virus antibody test remained negative. A graphical representation illustrating the clinical progression, treatment interventions, and disease activity over time is provided in Figure 4.

## 3 Discussion

The pathogenesis of SuS remains elusive. SuS has been associated with an autoimmune mechanism involving inflammatory mediators or autoantibodies, such as anti‐endothelial cell IgG1 antibodies (AECA) [14, 15], leading to microinfarcts in the retina, inner ear, and brain [16, 17]. In contrast, recent evidence suggests that autoreactive CD8^+^ cytotoxic T‐cells may play a central role in disease pathogenesis, inducing inflammation‐driven endothelial injury and micro‐ischemic damage [12]. This hypothesis was supported by a study demonstrating disease amelioration in a mouse model of SuS by inhibiting T cell‐endothelial adhesion, notably via very late antigen‐4 (VLA‐4) blockade by natalizumab. In this study, four patients with therapy‐refractory SuS showed reduced relapses, disease progression, and MRI‐detected CNS lesions after natalizumab treatment. However, the occurrence of relapses in two patients following the discontinuation of natalizumab suggests that while VLA‐4 blockade may reduce immune cell trafficking, it does not wholly abrogate the underlying disease process [12].

In this report, both female individuals presented with the complete triad of sensorineural hearing loss, retinal damage, and characteristic brain lesions, facilitating a straightforward diagnosis. Although the classic triad is pathognomonic for SuS, the absence or subtle presentation of these clinical features can complicate diagnosis [10]. Given the variable course and rarity of SuS, no prospective studies provide definitive treatment guidelines. Our treatment regimen—consisting of corticosteroid pulse therapy followed by natalizumab and other adjuvant therapies—resulted in prolonged reductions (16 and 22 months respectively) in both clinical and paraclinical disease activity and fewer relapses, without adverse effects.

We opted to initiate treatment with natalizumab instead of other existing therapies such as IVIG or cyclophosphamide, as commonly recommended in the guidelines [11] drawing on our extensive experience with MS patients who have been treated with this medication for several years and based on the rationale published [12]. Furthermore, natalizumab offers significant advantages regarding practicality and a more favorable adverse effect profile when compared to IVIG and cyclophosphamide, as long as the anti‐JC virus antibody status remains negative [18]. The treatment regimen was refined based on continuous MRI evaluations, ophthalmologic and ENT assessments, and clinical follow‐up examinations. The successful use of anti‐α4 integrin therapy, natalizumab, supports the hypothesis that CD8^+^ T cells promote endotheliopathy in SuS and suggests this pathway as a potential therapeutic target. TPE may be an effective therapy in refractory cases, as it removes inflammatory mediators and both IgM and IgG autoantibodies; however, it does not eliminate cytotoxic T‐cells and likely has no long‐lasting effects, making natalizumab a potentially more advantageous treatment option [19].

However, the small number of patients treated precludes definitive conclusions. It is important to note that the natural progression of SuS may involve a spontaneous reduction in relapse frequency, and in many cases, relapses may cease entirely [11]. This complicates the determination of whether the effects of natalizumab are indeed disease‐modifying or if the observed improvements are due to spontaneous remission. This distinction is particularly relevant given a previously published case report that noted worsening of clinical symptoms following natalizumab therapy in a suspected SuS patient [20]. However, unlike our patients, this case involved an incomplete form of SuS, with only two core features (encephalopathy and hearing loss). Increased relapse rates following treatment with certain immunotherapies, including alemtuzumab, interferon beta, and natalizumab, are not uncommon in patients with atypical CNS inflammatory conditions [21, 22, 23]. One proposed mechanism for this phenomenon is that natalizumab may disrupt immune homeostasis by reducing circulating regulatory T cells and increasing the number of pro‐inflammatory cytokine‐producing T cells, while also impairing the migration of regulatory natural killer cells from the periphery into the brain, potentially triggering disease relapses [20, 21]. However, the relapse observed in our patient following the extension of the natalizumab dosing interval further underscores the possible beneficial role of this therapy in SuS management. Therefore, systematic controlled studies appear warranted.

The duration of immunosuppression in SuS is particularly unknown, particularly considering the risk of the return of disease activity, sometimes above baseline (so‐called rebound) after discontinuing natalizumab. Therefore, no evidence‐based suggestion is possible concerning treatments once remission is achieved using natalizumab. However, alternative maintenance therapy, for example, with MMF, potentially combined with tacrolimus, may be an option, previously suggested by others for at least 2 additional years [10].

Our observations suggest that natalizumab may constitute a promising targeted treatment approach, complementing existing treatment strategies and holding potential in other diseases where CD8^+^ T cell‐mediated endothelial damage is a critical pathogenic factor.

## 4 Conclusion

SuS is a rare and complex disorder requiring a high degree of clinical suspicion for accurate diagnosis. It is marked by relapsing and remitting inflammatory episodes, with some patients achieving complete remission, though many experience chronic inflammation leading to significant neurological sequelae. Natalizumab may be a viable treatment option to control disease activity and prevent progression. Further research and prospective controlled clinical studies are warranted to better assess the long‐term efficacy of natalizumab versus the natural disease course when managing SuS.

## Author Contributions

**Agni M. Konitsioti:** writing – original draft, methodology, resources, writing – review and editing, conceptualization, investigation, project administration. **Rafael Grajewski:** writing – review and editing, resources, investigation. **Mark Schlamann:** writing – review and editing, resources, investigation. **Michael Schroeter:** writing – review and editing, resources, investigation. **Gereon R. Fink:** writing – review and editing, resources, investigation, supervision. **Clemens Warnke:** writing – review and editing, resources, conceptualization, methodology, investigation, project administration, validation, supervision.

## Ethics Statement

The authors have nothing to report.

## Consent

Written informed consent for publication was obtained. Patient consent forms for the case report, including the original and an English translation, are attached as PDF files (Patient_1 Consent‐to‐Disclose Form [Confidential].pdf, Patient_2 Consent‐to‐Disclose Form [Confidential].pdf, English Translation—Patient Consent‐to‐Disclose Form [Confidential]).

## Conflicts of Interest

A.M.K. has received a study grant from Novartis. C.W. has received institutional support from Novartis, Alexion, Sanofi Genzyme, Biogen, Merck, Janssen, Bayer and Roche. He has received personal honoraria for teaching lectures from Biontech, Medpoint Medizinkommunikations, F&U confirm, Privatinstitut für Klinikmanagement, The Royal College Of Physicians, and for consulting from Wuesthoff & Wuesthoff and Bristows LLP. R.G. declares no conflicts of interest. Ma.S. declares no conflicts of interest. Mi.S. declares no conflicts of interest. G.R.F. declares no conflicts of interest.

## Supporting information


Table S1.

## Data Availability

The data that support the findings of this study are available on request from the corresponding author. The data are not publicly available due to privacy or ethical restrictions.
