# Supplementary material for: Successful Natalizumab Treatment of Two Female Individuals With Susac Syndrome
Source: Eur J Neurol. 2025 Mar 3;32(3):e70103. doi: 10.1111/ene.70103 (PMC11875067; doi:10.1111/ene.70103)
Supplement: Supplementary file 1 — Table S1. [file ENE-32-e70103-s001.docx]

**Supplementary table.** Laboratory and cerebrospinal fluid findings of both patients at diagnosis prior to initiation of steroid therapy.

| **Laboratory data** | **Case Number 1. 25-old female individual with spastic-ataxic** **left hemiparesis** | **Case Number 2. 28-old female patient with hypoacusis and visual disturbances** |
| --- | --- | --- |
| Oligoclonal bands | Type 1 | Type 1 |
| Cerebrospinal fluid basic findings | Cell count: 7/µl (<5) Polymorphonuclear cells (relative): 0% Mononuclear cells (relative): 100% Erythrocytes: Negative Lactate: 1.4 mmol/L (1.1 - 2.4) Protein: 1.04 g/L (0.15 - 0.45) | Cell count: 2/µL (<5) Polymorphonuclear cells (relative): 0% Mononuclear cells (relative): 100% Erythrocytes: Negative Lactate: 1.9 mmol/L (1.1 - 2.4) Protein: 0.53 g/L (0.15 - 0.45) |
| Immunoglobulins (CSF and serum) | Immunoglobulin G (CSF): 133.00 mg/L Immunoglobulin A (CSF): 15.10 mg/L Immunoglobulin M (CSF): 1.25 mg/L Immunoglobulin G (Serum): 14.30 g/L (7.0 - 16.0) Immunoglobulin A (Serum): 3.17 g/L (0.7 - 4.0) Immunoglobulin M (Serum): 1.43 g/L (0.4 - 2.3) | - |
| Antinuclear antibodies (ANAs) | ANAs were detected at a titer of ≥1:320 but could not be assigned to any of the common ANA subtypes in the ANA differentiation (IgG immunoblot). | ANAs were detected at a titer of ≥1:320 but could not be assigned to any of the common ANA subtypes in the ANA differentiation (IgG immunoblot). |
| Anti-Neutrophile cytoplasmatic antibodies (ANCAs) | negative | negative |
| Immunofixation | no evidence of monoclonal gammopathy | no evidence of monoclonal gammopathy |
| JC-virus antibody test | negative | negative |
| Electrophoresis | normal | normal |
| Antibody responses in sera towards HIV1, HIV2, hepatitis B, hepatitis C, varicella zoster;  towards borrelia and syphilis, sera and CSF | negative | negative |
| Antibody responses in sera towards CMV, EBV, bartonella and toxoplasma | - | negativ |
| Antibody response in sera towards measles, mumps, rubella;  Quantiferon TB test | negative | - |
| Complete blood count (CBC) | Lymphocytes absolute (EB): 1.08 × 10⁹/L (1.26 - 3.35)  T-Lymphocytes (CD3) relative (EB): 77.1% (55 - 83) T-Lymphocytes (CD3) absolute (EB): 0.83 × 10⁹/L (0.70 - 2.10) T-helper cells (CD4) relative (EB): 32.7% (28 - 57) T-helper cells (CD4) absolute (EB): 0.35 × 10⁹/L (0.30 - 1.40) T-suppressor cells (CD8) relative (EB): 43.4% (10 - 39) T-suppressor cells (CD8) absolute (EB): 0.47 × 10⁹/L (0.20 - 0.90) CD4/CD8 ratio: 0.7 (0.6 - 2.8) B-Lymphocytes (CD19) relative (EB): 12.8% (6 - 19) B-Lymphocytes (CD19) absolute (EB): 0.14 × 10⁹/L (0.10 - 0.50) NK-cells (CD16+56) relative (EB): 9.6% (7 - 31) NK-cells (CD16+56) absolute (EB): 0.10 × 10⁹/L (0.09 - 0.60) Activated CD8+ (CD38+ on CD8+) relative (EB): 24.7% Activated CD8+ (CD38+ on CD8+) absolute (EB): 0.12 × 10⁹/L Activated T-cells (HLA-DR+ on CD3+) relative (EB): 25.3% (8 - 15) Activated T-cells (HLA-DR+ on CD3+) absolute (EB): 0.21 × 10⁹/L | Leukocytes: 15.78 × 10⁹/L (4.40 - 11.30) Erythrocytes: 4.72 × 10¹²/L (4.0 - 5.2) Hemoglobin: 13.9 g/dL (12.0 - 16.0) Hematocrit: 42% (36 - 45) MCV: 90 fL (80 - 96) MCH: 29 pg (28 - 34) MCHC: 33 g/dL (31 - 37) Erythrocyte Distribution Width: 12.3% (11.5 - 14.5) Platelets: 295 × 10⁹/L (150 - 400) MPV: 8.8 fL (7.2 - 11.1) Erythroblasts: 0.0/100 WBCs (0.0 - 0.0) Erythroblasts absolute: 0.0 × 10⁹/L Neutrophils relative: 89.6% (42.9 - 74.3) Neutrophils absolute: 14.15 × 10⁹/L (2.1 - 8.89) Immature Granulocytes relative: 1.2% (<1) Immature Granulocytes absolute: 0.19 × 10⁹/L Lymphocytes relative: 7.2% (18.3 - 45.7) Lymphocytes absolute: 1.13 × 10⁹/L (1.26 - 3.35) Monocytes relative: 1.8% (4.2 - 11.8) Monocytes absolute: 0.28 × 10⁹/L (0.25 - 0.84) Eosinophils relative: 0.1% (0.2 - 5.3) Eosinophils absolute: 0.01 × 10⁹/L (0.01 - 0.40) Basophils relative: 0.1% (0.1 - 1.0) Basophils absolute: 0.02 × 10⁹/L (0.01 - 0.07) |

**Legend:** pathological results are highlighted in red, with reference values provided in parentheses (min-max). Abbreviations: CMV: Cytomegalovirus, EBV: Epstein-Barr-Virus, TB: Tuberculosis
